# The Scavenger Receptor SSc5D Physically Interacts with Bacteria through the SRCR-Containing N-Terminal Domain

**DOI:** 10.3389/fimmu.2016.00416

**Published:** 2016-10-13

**Authors:** Catarina Bessa Pereira, Markéta Bocková, Rita F. Santos, Ana Mafalda Santos, Mafalda Martins de Araújo, Liliana Oliveira, Jiří Homola, Alexandre M. Carmo

**Affiliations:** ^1^i3S-Instituto de Investigação e Inovação em Saúde, Universidade do Porto, Porto, Portugal; ^2^IBMC – Instituto de Biologia Molecular e Celular, Porto, Portugal; ^3^ICBAS – Instituto de Ciências Biomédicas Abel Salazar, Universidade do Porto, Porto, Portugal; ^4^Institute of Photonics and Electronics of the Czech Academy of Sciences, Prague, Czech Republic; ^5^MRC Human Immunology Unit, Nuffield Department of Clinical Medicine, Weatherall Institute of Molecular Medicine, University of Oxford, Oxford, UK

**Keywords:** surface plasmon resonance, scavenger receptor cysteine-rich, pattern recognition receptors, bacteria

## Abstract

The scavenger receptor cysteine-rich (SRCR) family comprises a group of membrane-attached or secreted proteins that contain one or more modules/domains structurally similar to the membrane distal domain of type I macrophage scavenger receptor. Although no all-inclusive biological function has been ascribed to the SRCR family, some of these receptors have been shown to recognize pathogen-associated molecular patterns (PAMP) of bacteria, fungi, or other microbes. SSc5D is a recently described soluble SRCR receptor produced by monocytes/macrophages and T lymphocytes, consisting of an N-terminal portion, which contains five SRCR modules, and a large C-terminal mucin-like domain. Toward establishing a global common role for SRCR domains, we interrogated whether the set of five SRCR domains of SSc5D displayed pattern recognition receptor (PRR) properties. For that purpose, we have expressed in a mammalian expression system the N-terminal SRCR-containing moiety of SSc5D (N-SSc5D), thus excluding the mucin-like domain likely by nature to bind microorganisms, and tested the capacity of the SRCR functional groups to physically interact with bacteria. Using conventional protein–bacteria binding assays, we showed that N-SSc5D had a superior capacity to bind to *Escherichia coli* strains RS218 and IHE3034 compared with that of the extracellular domains of the SRCR proteins CD5 and CD6 (sCD5 and sCD6, respectively), and similar *E. coli*-binding properties as Spα, a proven PRR of the SRCR family. We have further designed a more sensitive, real-time, and label-free surface plasmon resonance (SPR)-based assay and examined the capacity of N-SSc5D, Spα, sCD5, and sCD6 to bind to different bacteria. We demonstrated that N-SSc5D compares with Spα in the capacity to bind to *E. coli* and *Listeria monocytogenes*, and further that it can distinguish between pathogenic *E. coli* RS218 and IHE3034 strains and the non-pathogenic laboratory *E. coli* strain BL21(DE3). Our work thus advocates the utility of SPR-based assays as sensitive tools for the rapid screening of interactions between immune-related receptors and PAMP-bearing microbes. The analysis of our results suggests that SRCR domains of different members of the family have a differential capacity to interact with bacteria, and further that the same receptor can discriminate between different bacteria strains and species.

## Introduction

Pattern recognition receptors (PRR) are membrane-bound or cytosolic receptors of plants and animals that are capable of interacting with pathogen-associated molecular patterns (PAMP), including lipopolysaccharide (LPS) of Gram-negative bacteria, the Gram-positive bacteria lipotheicoic acid (LTA) and peptydoglycan (PGN), as well as the fungi polysaccharides Zymosan or β-glucan, thus providing a first line of immune defense against microbes or their secreted toxins. Several families of PRR have been reported to be specific for pathogens or virulence factors, and they include Toll-like receptors, nucleotide-binding oligomerization domain (NOD)-like receptors, retinoic acid-inducible gene I (RIG-I)-like receptors, and C-type lectin receptors, among others (1). In contrast, receptors belonging to yet another group, the scavenger receptor cysteine-rich superfamily (SRCR-SF), are seldom referred to as pathogen-recognition molecules, despite the fact that several SRCR receptors have been shown to bind to and clear bacteria, fungi, or viruses from infected hosts (2).

Scavenger receptor cysteine-rich members are present in all animal phyla and although individual proteins may have various roles in, for example, cell differentiation, iron metabolism, homeostasis, or apoptosis, most SRCR proteins are thought to serve immune-related functions (3). A subfamily (group B) of the SRCR-SF consists of members present only in vertebrates (4), and four of the nine receptors described in humans have been shown to bind to bacteria or bacterial components. CD6 and CD163 are membrane-bound receptors of T cells and macrophages, respectively; DMBT1, which has a broad expression profile, and Spα, a soluble glycoprotein expressed by macrophages in the lymphoid tissues and highly present in the serum [detection levels of microgram per milliliter (5)], are molecular sensors of Gram-positive and Gram-negative bacteria (6–9). Although shown not to bind to either Gram-positive or -negative bacteria, the T cell surface SRCR protein CD5 is reported to interact with conserved fungal components and to aggregate fungal cells (10).

After bacterial challenges, the soluble SRCR protein Spα is immediately released from human macrophages to control bacteria spreading and inflammatory cytokine secretion by PRR-expressing innate cells (9). *In vivo* studies of PAMP-induced septic shock have shown that the levels of the Spα mouse homolog (mAIM/Api6/Cd5L) increase rapidly upon injection of LPS or Zymosan, further suggesting that this SRCR protein can act as a circulating PRR (11). SSc5D is a recently described soluble SRCR protein that shares many features with Spα. SSc5D is expressed in macrophages, T cells, and several epithelial cells, especially from placenta, spleen, and colon (12). It is also highly abundant in the serum and shows increased levels in inflammatory conditions (13). The mouse homolog of SSc5D [S5D-SRCRB (14)] is also upregulated upon infection and seems capable to bind bacteria (15), although this has not been reported for the human counterpart. A major difference between SSc5D and Spα relates not only to the number of SRCR domains (5 and 3, respectively) but also to the existence of a large mucin-like sequence located at the C-terminus of SSc5D. In the human molecule, this domain represents about 40% of the amino acid content of the whole protein, and it is expected that, similar to other *O*-glycosylated mucin-like proteins, it may bind and modulate pathogen behavior.

Label-free biosensors have revolutionized the qualitative and quantitative analysis of biomolecular interactions (e.g., protein–protein or protein–nucleic acids interactions) and are also broadly used in medical diagnostics, environmental monitoring, or food safety and security (16). Highly sensitive detection technology, such as surface plasmon resonance (SPR) that allows real-time studies of molecular binding processes, has been recently applied to the detection of bacteria and other microbial pathogens (17–19). These early studies have relied on the use of high-affinity antibodies recognizing particular components of bacterial surfaces. Despite the considerably weaker binding affinities for common receptor–ligand pairs when compared with antibody–antigen interactions, we hypothesized that an analogous strategy could be set up to scrutinize the interaction of secreted SRCR proteins with whole cell bacteria if these interactions were strong enough, reflecting a potential PRR nature of SRCR proteins.

In this work, we demonstrate the ability of SPR biosensor technology to monitor the interaction of secreted SRCR proteins with whole cell bacteria of different types. We have assessed the bacteria-binding capacity of the N-terminal moiety of SSc5D (excluding the mucin-like sequence likely to bind bacteria *per se*) and compared with the equivalent domains of other SRCR-family proteins, soluble Spα, and the extracellular domains of CD5 and CD6. The SPR experiments demonstrate the differential bacteria-binding capacity of N-SSc5D compared with the other SRCR proteins, and that globally these receptors can qualitatively distinguish between different types of bacteria.

## Materials and Methods

### Recombinant Protein Production and Purification

The soluble extracellular domain of CD6 (sCD6) was produced as previously described (20), and the remaining recombinant proteins (N-SSc5D, Spα, and sCD5) were expressed and purified as follows. A cDNA corresponding to the N-terminal half of SSc5D (exons 1–12), which includes the five SRCR domains (N-SSc5D) (12), was amplified by PCR from human placenta cDNA using forward 5′-TATAATGGATCCGAGCGCCTGCGCCTGGCCGAT and reverse 5′-AATAGGATCCCTCTTGTGTCCGGCAGGCGCCTTATTGCTGG primers (*Bam*HI restriction sites are underlined). The Spα cDNA was amplified by PCR from human spleen cDNA using forward 5′-TTAGGATCCTCTCCATCTGGTGTGCGGCTG and reverse 5′-CAAGGATCCACCTGAGCAGATGACAGCCAC primers. A cDNA fragment encoding the extracellular domain of human CD5 (residues Arg^25^-Ser^348^; sCD5) was amplified by PCR from a template CD5-pGFP-N1 kindly provided by G. Bismuth (Institut Cochin, Paris) using forward 5′-TAGGGATCCCGGCTCAGCTGGTATGAC and reverse 5′-CTAGGATCCCGGGGTTTGGATCTTGGCAT primers. Each cDNA was inserted into the *Bam*HI sites of the lab-modified version of pEE14 in order to obtain chimeric cDNAs encoding, in the following order, signal peptide, HA-tag, the specific protein sequence (Spα, N-SSc5D, or sCD5), a BirA recognition sequence, and 6·His tag sequences.

The sCD5, N-SSc5D, and Spα vectors were transfected into CHO-K1 cells using Lipofectamine (Invitrogen). Clones resistant to 30-μM methionine sulfoximine (MSX) were selected (21) and screened for soluble CD5 (HA-sCD5-BirA-His), N-SSc5D (HA-N-SSc5D-BirA-His), and Spα (HA-Spα-BirA-His) expression using dot blots and western blots. The best clones expressing HA-sCD5-BirA-His, HA-N-SSc5D-BirA-His, and HA-Spα-BirA-His were selected for large-scale production of protein and grown in cell factories (Nunc). Proteins secreted into tissue culture supernatants were harvested after approximately 4 weeks and purified by metal-chelate chromatography using Ni Sepharose High Performance (HisTrap HP, GE Life Sciences). HA-sCD5-BirA-His was eluted from the Ni column with 250 mM imidazole in PBS, while HA-N-SSc5D-BirA-His and HA-Spα-BirA-His were eluted with 40 mM imidazole in PBS. Fractions containing the HA-N-SSc5D-BirA-His and HA-Spα-BirA-His were further purified by anionic chromatography (UNO Q column BioRad) with 1 M NaCl. The previously produced recombinant protein sCD6 also conformed to a similar structure as the newly produced proteins, having a HA-sCD6-BirA-His sequence.

Protein purity was analyzed by SDS-PAGE (Figure S1 in Supplementary Material). Samples of the fractions obtained by chromatography were run for 1 h at 150 V, and the gels were stained with Bio-Safe Coomassie Premixed Staining Solution (Bio-Rad Laboratories) for visualization of the protein products.

For N-SSc5D immunoblotting, samples were run in SDS-PAGE for 1 h at 150 V with Tris/glycine/SDS running buffer (Bio-Rad Laboratories). Samples were transferred to the nitrocellulose membrane using the iBlot™ Gel Transfer Device (Invitrogen) following the manufacturer’s instructions. Then, the membrane was blocked with TBS, 0.1% Tween 20 (TBS-T), containing 5% non-fat dried milk, for 1 h with shaking. N-SSc5D was subsequently detected with rabbit anti-SSc5D (Abgent, 1:5,000) primary antibody in TBS-T with 3% non-fat dried milk, for 1 h at RT, followed by peroxidase-conjugated goat anti-rabbit IgG antibody (Sigma, 1:30,000) for 1 h at RT. The immunoblot was developed using ECL detection reagent (GE Healthcare Life Sciences), and the image was acquired in a ChemiDoc XRS+ system (Bio-Rad Laboratories).

### Bacteria Strains

*Listeria monocytogenes* EGD-e was grown in brain heart infusion (BHI) medium (BD-Difco) at 37°C to an optical density of 0.6 at 600 nm (OD_600_; exponential phase), and *Escherichia coli* strains [BL21(DE3), IHE3034, RS218] were grown in LB medium at 37°C to an OD_600_ of 0.45.

### Conventional Bacteria–Protein Binding Assays

Recombinant proteins Spα, N-SSc5D, sCD6, and sCD5 (5 μg per assay) were incubated for 1 h at 4°C with the indicated cell suspensions of live bacteria (1 × 10^8^ cells) in binding buffer (TBS, 1% BSA, 5 mM CaCl_2_). Suspensions were centrifuged at 4,000 × *g* for 5 min at 4°C. Cell pellets were washed thoroughly, then resuspended in 40-μl Laemmli’s sample buffer, and denatured by heating at 95°C for 10 min. Next, 20 μl of this lysate and pure recombinant proteins (25 or 100 ng) were separated in 6% SDS-PAGE. The gel was run for 1 h at 150 V with Tris/glycine/SDS running buffer (Bio-Rad Laboratories). After the SDS-PAGE, proteins were transferred to the nitrocellulose membrane using the iBlot™ Gel Transfer Device (Invitrogen) following the manufacturer’s instructions. Then, the membrane was blocked with TBS-T containing 5% non-fat dried milk, for 1 h. Cell-bound protein was subsequently detected using mouse IgG1 anti-HA (clone 16B12) from Covance (0.1 μg/ml) in TBS-T with 3% non-fat dried milk, for 1 h at RT, followed by goat anti-mouse HRP-conjugated (Santa Cruz Biotechnology) (0.02 μg/ml) in the same conditions. The immunoblot was developed using ECL detection reagent (GE Healthcare Life Sciences), and the image was acquired in a ChemiDoc XRS+ system (Bio-Rad Laboratories).

### SPR-Based Detection of Whole Bacterial Cell Interaction with SRCR Proteins

We used a laboratory four-channel SPR platform based on the wavelength spectroscopy of surface plasmons (Plasmon IV) (22) developed at the Institute of Photonics and Electronics, Czech Republic. In this SPR biosensor, the sensor response is expressed as a shift in the wavelength of SPR resonance and is directly proportional to the mass of biomolecules attached to the surface of the sensor. Using the calibration procedure described in Ref. (23), the surface density of both the immobilized receptors and the subsequently attached molecules can be determined. For an SPR resonance of around 750 nm, the shift of 1 nm in the SPR wavelength represents a change in the protein surface coverage of 17 ng/cm^2^ (23). All the experiments were performed at 25°C. Buffers used were SA_10_ (10 mM sodium acetate, pH 4.0/5.0), PBS (10 mM phosphate, 2.9 mM KCl, 137 mM NaCl, pH 7.4), PBNa (10 mM phosphate, 2.9 mM KCl, 750 mM NaCl, pH 7.4), and Tris (10 mM Tris-HCl, pH 7.4).

### Functionalization of the Sensor Chip

The sensor chip was functionalized with a mixed self-assembled monolayer (SAM) by incubating the cleaned gold chip in degassed absolute ethanol with a mixture (7:3) of HSC_11_(EG)_4_OH and HSC_11_(EG)_6_OCH_2_COOH alkanethiols at a final concentration of 200 μM. The HSC_11_(EG)_6_OCH_2_COOH alkanethiols terminated with a carboxylic head group were used to anchor a receptor by amino coupling, while HSC_11_(EG)_4_OH alkanethiols terminated with hydroxylic group were used to form a stable non-fouling background. For that purpose, the sensor chip was immersed in a mixed thiol solution at a temperature of 40°C for 10 min and then stored overnight in the dark at RT. After the formation of the mixed SAM, the chip was removed from the solution, rinsed with absolute ethanol and deionized water, and dried with nitrogen. The chip was then immediately mounted to the prism on the SPR sensor. The activation of carboxylic terminal groups was performed *in situ* by injecting deionized water followed by a 1:1 mixture of NHS and EDC for 5 min and deionized water again.

Conditions for immobilization have been optimized in terms of running buffer composition and pH, as well as sufficient surface coverage. Immobilization of proteins *via* covalent attachment to COOH/OH SAM was performed at a flow rate of 30 μl/min and a temperature of 25°C. To immobilize the receptors, sodium acetate (SA_10_) pH 4.0 (Spα, N-SSc5D, and sCD6) or 5.0 (sCD5) was flowed trough the activated surface until a baseline was achieved. Then, the SA_10_ solutions containing the receptors (2–5 μg/ml) were flowed across the activated surface until a desired surface coverage was achieved. To remove the non-covalently bound receptors, the high ionic strength PBNa buffer was flowed along the sensor surface. Finally, the sensor surface was treated with 1 M EA to deactivate residual carboxylic groups.

### Detection of the Interaction of SRCRs with Bacteria

Bacteria cells were pelleted by centrifugation (4,000 × *g*, 5 min) and resuspended in PBS. Then, to preserve bacterial cell morphology and to increase the sensitivity of the detection, cell aliquots were exposed to isopropanol (final concentration, 70% v/v) for 20 min at RT. The pellets of isopropanol-fixed cells were obtained by centrifugation at 7,000 × *g* for 5 min and washed twice with PBS. Next, running buffer was flowed along the sensor surface until the baseline was achieved. Bacteria were resuspended in the running buffer at a concentration of 1 × 10^7^ CFU/ml (or as indicated in the text) and delivered at a flow rate of 50 μl/min to the sensor surfaces with the immobilized proteins. Then, the running buffer was introduced again. The binding of bacteria to the sensor surface was detected as the difference in the sensor response between the equilibrium level after washing the bound surface with the running buffer and the baseline level obtained before the injection of the bacteria solution.

In this work, we used reference-compensated measurements and tested several different surfaces to be used as a reference surface. These included a surface without receptors, surfaces covered with blocking molecules such as BSA, casein, or NeutrAvidin, and a surface with immobilized reference protein (sCD5). The study revealed that there was considerable adsorption of bacteria to a bare alkylthiolate SAM (used as a functional layer) without any receptors/molecules immobilized and that the binding of bacteria to the surface coated with blocking molecules was significantly higher than that observed in case of surface coated with a reference protein. Therefore, this approach was selected as the best option.

## Results

### Detection of N-SSc5D Binding to Bacteria in Conventional Bacteria–Protein Binding Assays

We first assessed the binding of the SRCR-containing extracellular domains of Spα, SSc5D, CD6, and CD5 (respectively, Spα, N-SSc5D, sCD6, and sCD5) to *E. coli* strains BL21(DE3), IHE3034, and RS218, and to *L. monocytogenes* strain EGD-e, using conventional bacteria-protein binding assays. Although Spα, sCD5, and sCD6 had previously been tested for bacteria binding (6, 9, 10), no experiments had been performed for SSc5D. We incubated 5 μg of each recombinant SRCR protein with bacterial suspensions of 1 × 10^8^ live cells (colony-forming units, CFU) at 4°C, followed by centrifugation and immunoblotting of the pelleted bacteria.

**Figure 1:**
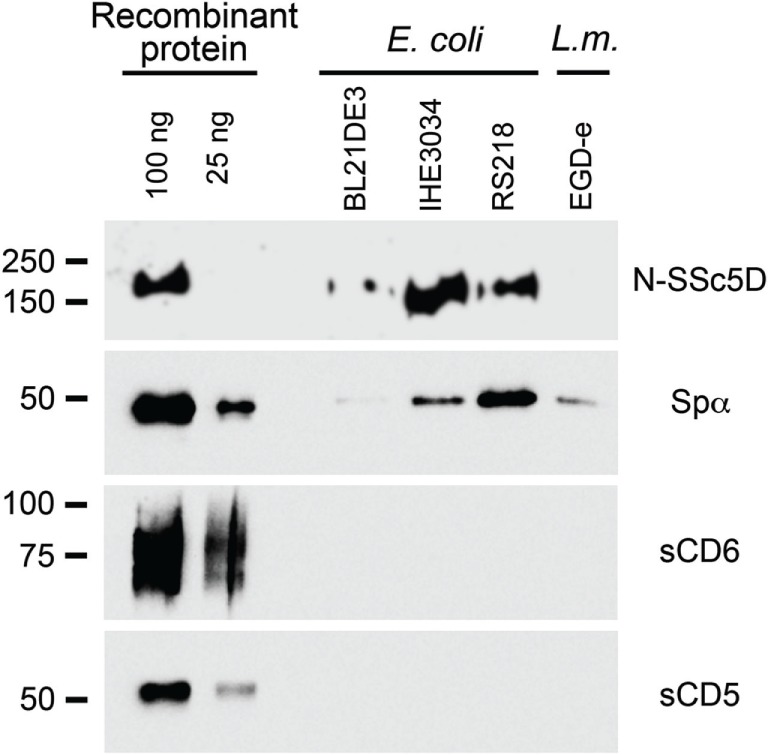
**Spα and N-SSc5D bind bacteria**. Recombinant Spα, N-SSc5D, sCD6, and sCD5 were incubated (5 μg each sample) with suspensions of 1 × 10^8^ CFU of live *E. coli*, strains BL21(DE3), IHE3034, or RS218, or with *L. monocytogenes*, strain EGD-e. Cell-bound proteins were detected by immunoblotting using anti-HA mAb. Pure recombinant proteins were also run (100 and 25 ng, left lanes) to determine the sensitivity of the assay.

We confirmed the interaction of recombinant Spα with all bacterial samples tested, having an enhanced capacity to bind *E. coli* RS218 comparing with the other bacteria strains (Figure 1). However, and in contrast with previous studies, no detectable sCD6 was recovered in association with the bacterial pellets, using our experimental setup. There was also no bacteria-bound sCD5 detected, but this was expected, given that CD5 was reported not to bind to bacteria (10). As observed from the experiments, N-SSc5D distinctly detected *E. coli* RS218 and IHE3034, although there was no conclusive evidence at this stage that it could bind to *E. coli* BL21(DE3) or to *L. monocytogenes*.

### N-SSc5D and Spα Binding to Bacteria Is Measurable by SPR

The results from the previous experiment suggested that different SRCR proteins had distinct binding properties to different bacterial strains, which might not have been highlighted in previous publications, each addressing a different SRCR protein at a time. Aware that western blot detection might not be the most sensitive method to emphasize these differences, we designed a new SPR-based assay to enhance the sensitivity of detection of extracellular proteins binding to bacteria. In this assay, the proteins are directly attached to the sensor chip by amine coupling. Suspensions of isopropanol-fixed bacteria, resuspended at a concentration of 1 × 10^7^ CFU/ml or lower, are then delivered to the sensor surface containing the immobilized proteins. The output of the SPR sensor (expressed in nanometer of resonant wavelength) is directly proportional to the amount of biomolecules attached to the active surface of the sensor. The difference in the sensor output before injection of bacteria (baseline level) and after washing the surface with captured bacteria in buffer is therefore proportional to the amount of bacteria captured (irreversibly bound) by the proteins immobilized on the sensor surface. This quantity was used to characterize the ability of the respective immobilized proteins to bind bacteria.

**Figure 2:**
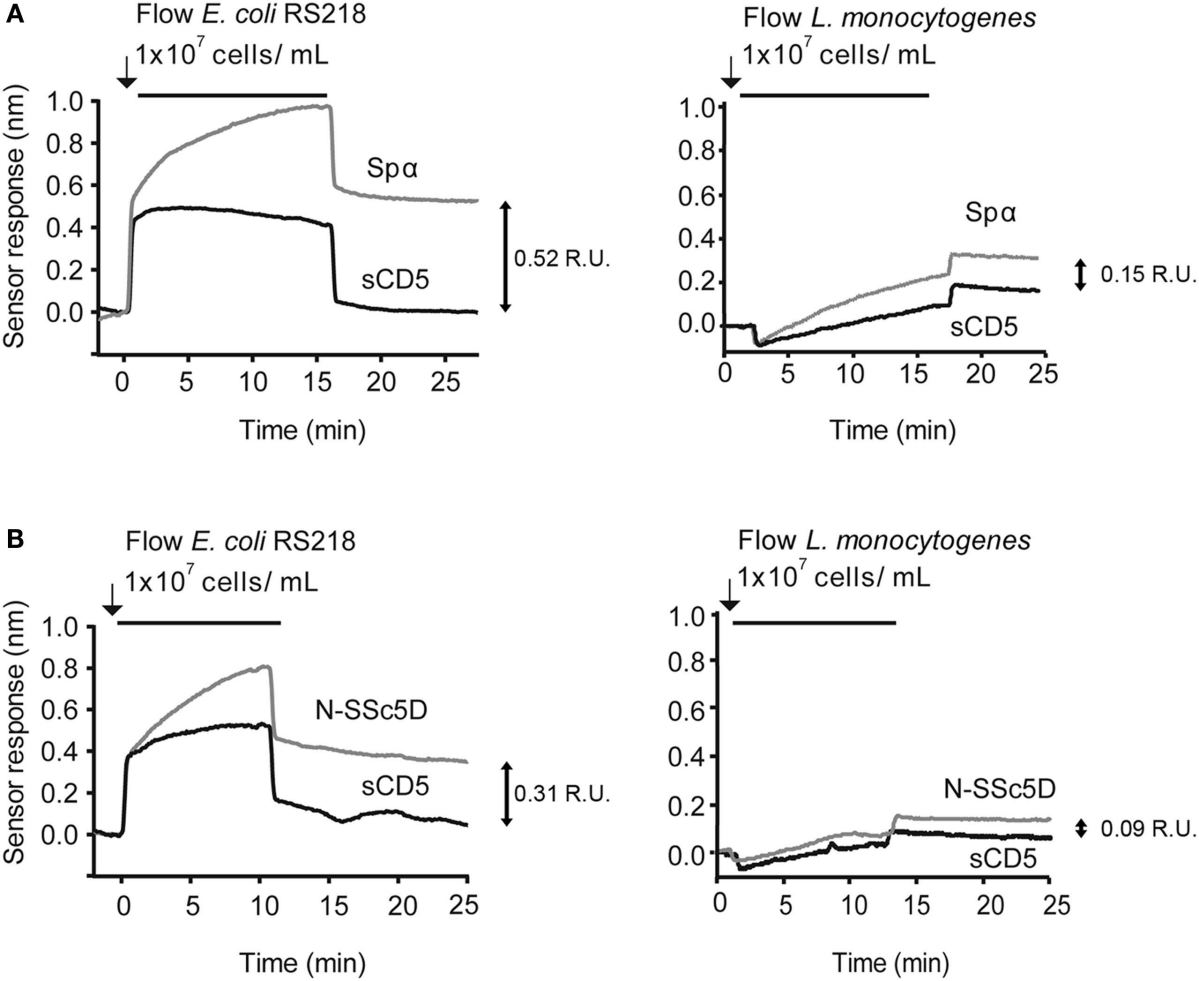
**SPR detection of N-SSc5D binding to *E. coli* RS218 and *L. monocytogenes* EGD-e**. Recombinant Spα **(A)** or N-SSc5D **(B)**, as well as the control sCD5 were immobilized in sensorchips and flowed with *E. coli* RS218 (left) or *L. monocytogenes* EGD-e (right) suspensions of 1 × 10^7^ CFU/ml. After injection stopped, bacteria were retained in the different surfaces containing the SRCR proteins according to the strength of binding. Data are representative of multiple experiments with similar results. R.U., response units.

We considered the following as references for the binding spectra: (a) the positive interaction of Spα with neuropathogenic *E. coli* K1 RS218 and with *L. monocytogenes* EGD-e and (b) the null interaction of sCD5 with both bacteria species (Figure 2A). Spα and sCD5 were immobilized in alternate flow chambers, bacteria were injected at 1 × 10^7^ CFU/ml, and SPR plots registered.

Next, we tested whether the interactions of N-SSc5D with *E. coli* RS218 and *L. monocytogenes* EGD-e were measurable by SPR. As illustrated in Figure 2B, the interaction levels of N-SSc5D with bacteria were lower than those of Spα in both cases (between 15 and 40% across several experiments), but quite distinct from the profiles obtained for sCD5. These results confirmed the WB detection of the N-SSc5D-*E. coli* RS218 interactions seen in Figure 1, but further advanced in the detection of a subtle interaction between N-SSc5D with *L. monocytogenes*.

The results were reliable and qualitatively consistent among experiments, with only small variations in the absolute values of the responses. The chip-to-chip reproducibility of the interaction was >82% and >95% for N-SSc5D and sCD5 binding, respectively. The reproducibility values were determined from three independent experiments for each protein.

### N-SSc5D Can Distinguish between Bacterial Strains

**Figure 3:**
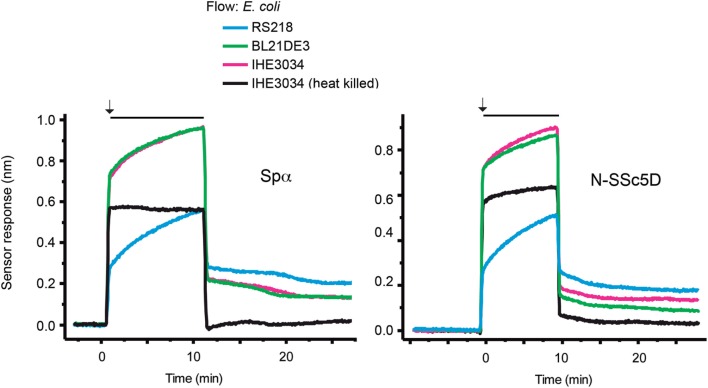
**Temporal sensor response to the differential binding of N-SSc5D and Spα to different *E. coli* strains**. Recombinant N-SSc5D (and Spα in parallel experiments) was immobilized in the four sensing channels and simultaneously injected suspensions of 1 × 10^7^ CFU/ml of *E. coli* RS218, IHE3034, or *E. coli* BL21(DE3). The fourth flow channel was used to flow heat-killed IHE3034. After 10 min of injection, bacteria were differently retained in the four different sensor chambers. Data are representative of multiple experiments with similar results.

To test whether N-SSc5D could have a different capacity to bind different *E. coli* strains, we immobilized N-SSc5D and simultaneously injected, in separate flow channels, the non-pathogenic laboratory BL21(DE3) strain, and the meningitis-causing RS218 and IHE3034 *E. coli* strains. As another control of null-binding, we used in the fourth flow channel, heat-killed IHE3034. In parallel, we performed the same experiment with immobilized Spα. As seen in Figure 3, *E. coli* RS218 gave the best binding curve to N-SSc5D, followed by IHE3034, and finally BL21(DE3). Heat-killed IHE3034 only marginally bound to N-SSc5D, suggesting that the bacterial determinants recognized by N-SSc5D are destroyed by heat. The binding profile of Spα to the different *E. coli* strains was not too different, binding marginally better to RS218 and BL21(DE3) than N-SSc5D, and less to IHE3034 than N-SSc5D, indicating that these proteins have slightly distinct recognition profiles but can nevertheless distinguish between different bacterial strains.

### Differential Binding of SRCR Proteins to a Same Bacterial Strain

**Figure 4:**
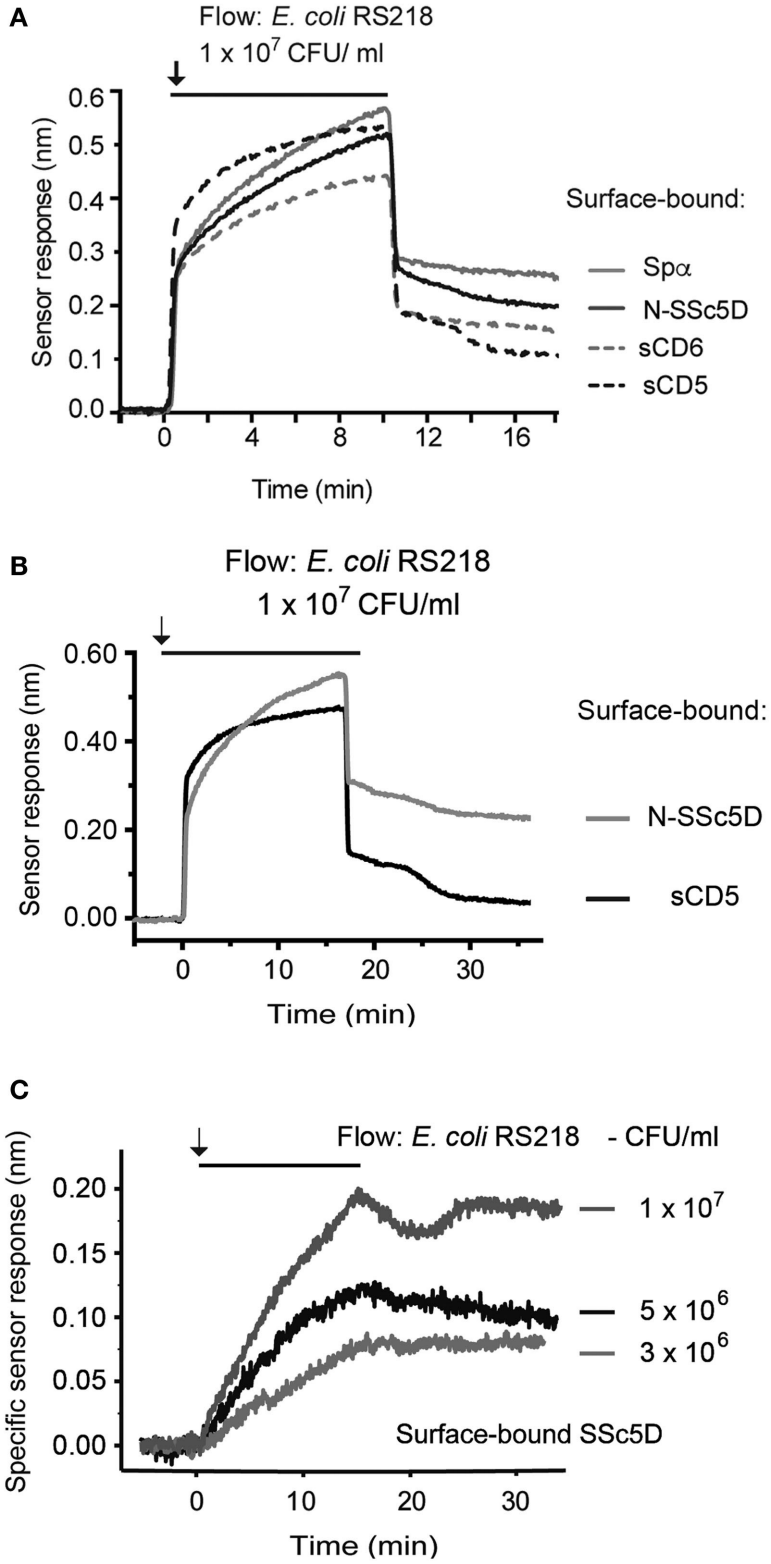
**Temporal sensor response to the binding of SRCR proteins to *E. coli* RS218**. **(A)** Recombinant Spα, N-SSc5D, sCD6, and sCD5 were immobilized in the four sensing channels and simultaneously injected with an *E. coli* RS218 suspension of 1 × 10^7^ CFU/ml. **(B)** Recombinant N-SSc5D and sCD5 were immobilized on alternate chambers, and *E. coli* RS218 was flowed at 1 × 10^7^ CFU/ml. **(C)** The specific binding of *E. coli* RS218 to N-SSc5D was obtained by the subtraction of the non-specific response registered for sCD5 from the measured signals of *E. coli* RS218 binding to N-SSc5D, for bacterial concentrations of 3, 5, and 10 × 10^6^ CFU/ml.

To directly assess the differential binding capacity of the different SRCR receptors to a same bacterial preparation, we immobilized Spα, N-SSc5D, sCD6, and sCD5 in the four sensing channels and simultaneously injected *E. coli* RS218 at 1 × 10^7^ CFU/ml to all channels. As depicted in Figure 4A, RS218 bound with the highest level to Spα, followed by N-SSc5D. As expected, sCD5 displayed the lowest level of RS218 binding; however, binding of the bacteria to immobilized sCD6 was, although relatively low, noticeably higher than that binding to sCD5. This indicates that despite the apparent negative result of Figure 1, there is some above-background level of binding of sCD6 to *E. coli* RS218 measurable by this SPR-based method.

Finally, we evaluated the sensitivity of the method by analyzing the interaction of *E. coli* RS218 with N-SSc5D using suspensions with decreasing bacteria concentration. Figure 4B represents again the profiles of binding of *E. coli* RS218 at 1 × 10^7^ CFU/ml to immobilized N-SSc5D and sCD5. Then, the specific binding was obtained by subtracting the signals arising from the measuring channels with immobilized N-SSc5D from those measured in the sCD5-immobilized reference channels. Three different concentrations of bacteria were used, 3, 5, and 10 × 10^6^ CFU/ml, and for each concentration, the subtractive plots are represented in Figure 4C, indicating that the method clearly detects specific binding of *E. coli* RS218 to N-SSc5D even when using bacteria concentrations as low as 3 × 10^6^ CFU/ml.

## Discussion

The SRCR-B family comprises a group of proteins that have a very high level of genetic conservation and remarkable structural similarity of the SRCR domains. However, each member has been described with very exclusive functions, as diverse as roles in signal transduction, regulation of inflammation, cell survival and apoptosis, differentiation, detoxification in iron metabolism, to name just a few, to such an extent that the structural properties of the SRCR modules may be so far the only proven unifying feature of the family. This diversity in functions can be in part explained from the fact that each protein has unique features (different number of SRCR domains), is expressed in different contexts and architectures (membrane bound in different cell types, carrying cytoplasmic domains of variable lengths and compositions, or is secreted), may have additional domains of other types, and can display different degrees of posttranslational modifications, such as *O*- and/or *N*-glycosylation.

Recently, the description of a physical interaction between Spα, which is a small soluble protein almost exclusively composed of the three SRCR domains, and several strains of bacteria (9) projected an explicit PRR function for such type of domain. Similar microbe-binding properties of other SRCR proteins have indeed been assigned to their own SRCR domains (6–8). To further explore this possible unifying role for SRCR domains, we thus investigated the PRR-type properties of the recently described protein SSc5D, and more specifically of its SRCR-containing moiety. For this purpose, we designed an SPR-based assay for rapid and direct detection of immune receptor–bacteria interactions.

Conventional methods used previously to assay the interaction of bacteria with secreted recombinant SRCR (or other) proteins, such as flow cytometry or immunoblotting, rely on the labeling of proteins with a fluorescent dye, such as FITC (24), or with biotin targeting the sulfhydryl groups of cysteine residues (6, 9, 10). Among the many practical advantages of the SPR method compared with conventional ones, there is no requirement for receptor labeling, and only minute amounts of protein are needed to generate distinct or differential signals. In our conventional assays shown in Figure 1, we used 5 μg of recombinant protein and 1 × 10^8^ CFU per individual receptor–bacteria assay, and some of these interactions were on the borderline of western blot sensitivity. By comparison, 2 μg of recombinant protein could be used in a single SPR assay testing the interaction with up to four bacteria types, these also used at smaller amounts (typically at 1 × 10^7^ CFU/ml, but feasibly down to 3 × 10^6^ CFU/ml), which represent an improvement of the detection of protein–bacteria interactions. Moreover, the versatility of our setup allows having up to four different immobilized proteins and simultaneously comparing the binding of each protein to the same bacterial suspension as analyte, or conversely, comparing directly in the same assay suspensions of four different bacteria binding to the same immobilized protein.

Surface plasmon resonance biosensor technology-based affinity and kinetic measurements are typically performed with analytes that are monovalent (25). Although through complex analyses it is possible to obtain such parameters in the case of multivalent (bacterial) contacts (26), we have utilized SPR to detect interaction *per se* and to make synchronized measurements, obtaining direct comparable data for sets of four different receptors, or four different bacteria samples. Detection of binding of bacteria to macromolecules, including lipids and carbohydrates, has been accomplished before (26, 27), but to the best of our knowledge, this is the first SPR study addressing the interaction between a host PRR and bacteria. It should be noted that we chose to consider the amount of captured (irreversibly bound) bacteria to characterize the ability of the respective proteins to bind selected bacteria, as the reported experiments with bacteria are complex, and the binding curves in response to bacteria are not determined only by kinetic parameters of the interactions; they are also affected by other factors, such as background refractive index changes (due to differences in the composition of samples containing bacteria and running buffer), the non-specific adsorption of bacteria, or other non-target molecules onto the sensing surface and mass transport (due to rather slow diffusion of bacteria to the sensing surface).

From the experiments described in the present work, we show for the first time that, like some other human SRCR proteins, SSc5D, through its set of SRCR domains, has the capacity to bind bacteria and, from the direct comparisons established using the multichannel SPR apparatus, that N-SSc5D and Spα can distinguish between different types of bacteria on one hand and different strains of one type of bacteria on the other. Binding of N-SSc5D and Spα to *E. coli* RS218 gave higher sensor responses than binding to BL21(DE3). While BL21(DE3) is a well-characterized non-pathogenic research model commonly used in academic laboratories and in the biotech industry, RS218 is a pathogenic strain belonging to the serotype O18ac:H7:K1 and displaying virulence factors that contribute to the onset of meningitis. The IHE3034 strain also belongs to the same serotype and although N-SSc5D binds better to IHE3034 than to BL21(DE3), the same behavior is not observed for Spα, suggesting that SRCR proteins may have very defined discriminatory properties on different, still undefined, extracellular components of bacteria. Likewise, the response signals for N-SSc5D and Spα binding to *L. monocytogenes* were significantly lower than to *E. coli*, possibly reflecting a differential sensing of Gram-positive vs. Gram-negative bacteria, but at this stage and with very few bacteria types tested, it is premature to establish any categorization.

The interactions of N-SSc5D and Spα with *E. coli* RS281 were relatively strong and specific and, as shown for N-SSc5D, the sensor responses increased proportionally to the concentration of the bacterial suspensions used. Comparing with the conventional assays, binding to *E. coli* IHE3034, also a meningitis-causing pathogen, did not give the same precise results, as N-SSc5D bound less and Spα bound better in the SPR experiments than in the bacteria-binding assays. SPR offers substantial benefits when compared with these methods, because it allows real-time detection of bacteria and, moreover, since bacteria are delivered under conditions of continuous hydrodynamic flow, the SPR technique is expected to better mimic the protein–bacteria interaction under physiological conditions where shear forces promoted by the body fluids are likely present (28, 29). As measurements are obtained simultaneously for the different proteins/bacteria within the same experiment, we can be confident that they truly reflect quantitative differences in binding of SRCR proteins to bacteria.

An important aspect in the design of the assay is the choice of a reference, which allows for the compensation of the binding of non-target molecules to the sensing surface. In the context of our study, sCD5 was defined as such based on the literature and on the result obtained with our conventional assay. Additionally, we chose to use sCD5 in experiments, as this protein is genetically and structurally related with the query molecules N-SSc5D and Spα, and thus it would account for intrinsic unspecific binding features that can be particular to the SRCR family of molecules.

CD6, on the other hand, was reported to bind to Gram-positive and Gram-negative bacterial strains (6). CD6 is a receptor of T lymphocytes that has characterized roles in the regulation of T cell signaling and in inflammatory responses (20, 30), so its role as a pathogen sensor was unexpected. From the results of our conventional assay shown in Figure 1, we would have concluded that either sCD6 does not bind to the tested bacteria or that it binds with such low affinity that the interaction does not survive the pelleting and washing of the bacteria. The lack of binding could not be attributed to any functional defect of our produced sCD6 protein, as this was shown to clearly bind its natural ligand CD166 by flow cytometry (Figure S2 in Supplementary Material). However, our improved SPR assays may highlight a slightly different conclusion: although the level of binding of sCD6 to *E. coli* RS218 (Figure 4) or to *L. monocytogenes* (data not shown) was significantly lower than that of either N-SSc5D or Spα, it stayed clearly above the level of the sCD5 negative profile. Apart from the higher sensitivity over the previous methods, SPR is run at the more adequate temperature of 25°C, whereas conventional protein–bacteria binding assays are customarily performed at 4°C. Notwithstanding the fact that the bacteria-binding capacities of sCD6 are reduced comparing with N-SSc5D or Spα, it is nonetheless very plausible that sCD6 may have true microbe-sensing properties, which are highlighted by its capacity to protect animals from LPS-induced septic shock (6).

In conclusion, we have demonstrated through the use of a dynamic, antibody-free, SPR-based assay that N-SSc5D, like Spα, is capable to physically interact with whole bacteria cells. This new approach can be adapted to screen for interactions with a wide range of bacteria and once the best bacterial targets of N-SSc5D are identified, this will hopefully allow to better characterize and more deeply explore the role of this SRCR protein in pathogen sensing and in driving immune responses. The results obtained in this study using the SRCR-containing moiety of SSc5D will undoubtedly further our understanding of the specific function of SRCR domains as the functional parts of a family of mammalian proteins that have enhanced capabilities to recognize and eventually fight bacterial pathogens.

## Author Contributions

CP designed and produced recombinant proteins, executed SPR experiments, and wrote the paper; MB designed and executed SPR experiments and wrote the paper; RS produced recombinant proteins and bacteria strains; AS and MA designed and produced recombinant proteins; LO performed experiments with bacteria strains; JH designed the SPR experiments and wrote the paper; AC planned and designed the study and wrote the paper.

## Conflict of Interest Statement

The authors declare that the research was conducted in the absence of any commercial or financial relationships that could be construed as a potential conflict of interest.

## Supplementary Material

Figure S1 **Proteins purified and used in SPR assays**. **(A)** Coomassie-staining of sCD5, sCD6, and Spα proteins purified from TCS with Ni-NTA resin and imidazole elution. Gels of sCD6 and sCD5 (12%) and Spα (10%) were run under reducing conditions. Identity of the proteins was confirmed by immunoblotting (not shown). **(B)** Anionic chromatography of N-SSc5D recovered from TCS samples and further purification. **(C)** N-SSc5D fractions 43–46 were collected and run on 7.5% SDS-PAGE and stained with Coomassie. N-SSc5D expression was confirmed by immunoblotting (bottom).

Figure S2 **sCD6 binds to cells expressing its ligand, CD166**. Panel of cells screened using recombinant sCD6 tetramers for binding to CD166. Cells were incubated with streptavidin-PE as an isotype control (red), sCD6 tetramers (blue), and sCD58 tetramers (green). **(A)** Jurkat and K562 cells do not express CD166, and therefore sCD6 tetramers showed no binding. sCD58 tetramers bind to CD2, which is highly expressed at the surface of the Jurkat T cell line. **(B)** The THP-1 (monocytic) and Raji (B cell) lines express CD166 at their surface (but not CD2) and therefore displayed binding of the sCD6 tetramers.
