# Supplementary figures and images for: The Scavenger Receptor SSc5D Physically Interacts with Bacteria through the SRCR-Containing N-Terminal Domain
Source: Front Immunol. 2016 Oct 13;7:416. doi: 10.3389/fimmu.2016.00416 (PMC5061727; doi:10.3389/fimmu.2016.00416)

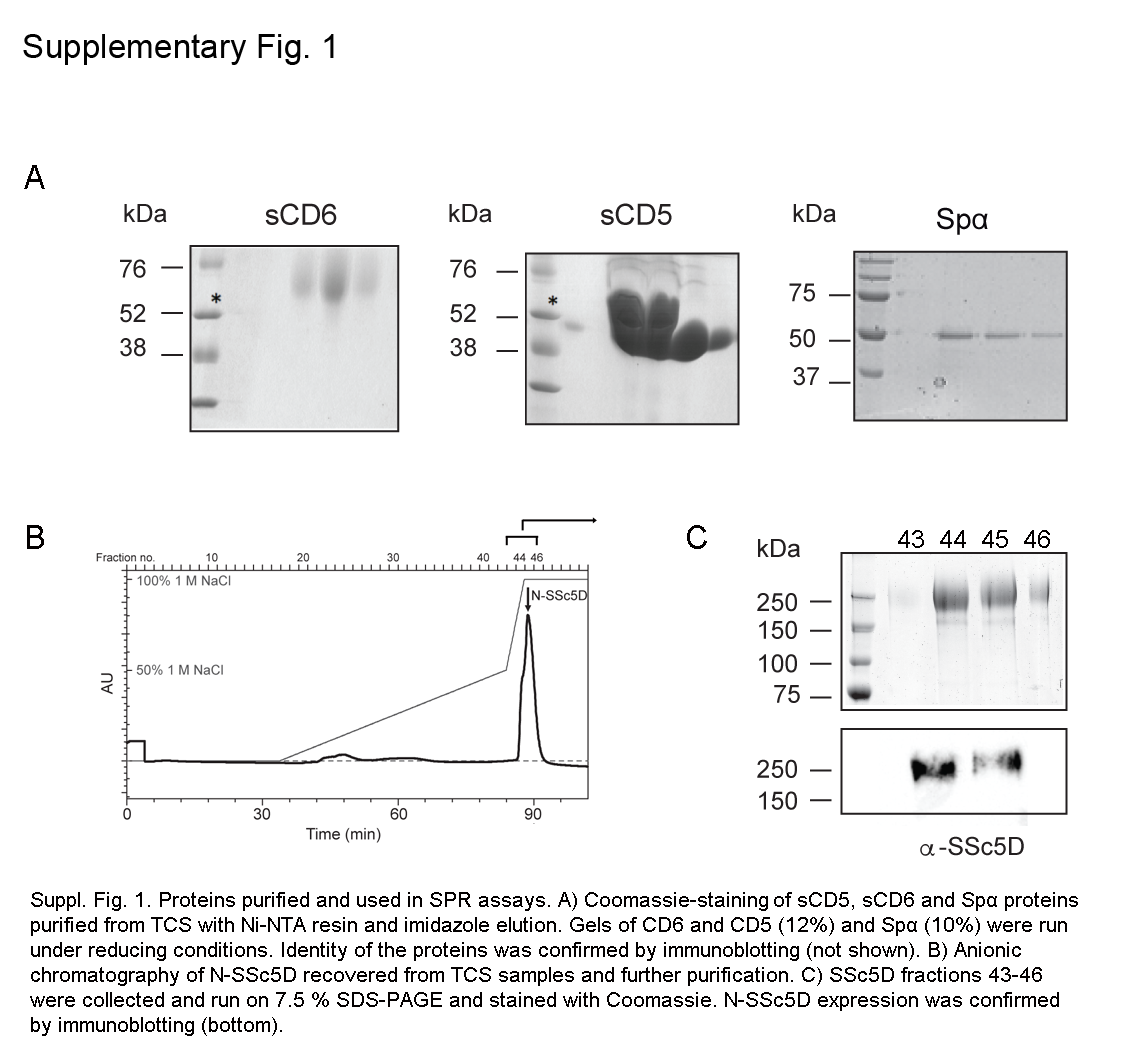

Supplement: Figure S1 — Proteins purified and used in SPR assays. (A) Coomassie-staining of sCD5, sCD6, and Spα proteins purified from TCS with Ni-NTA resin and imidazole elution. Gels of sCD6 and sCD5 (12%) and Spα (10%) were run under reducing conditions. Identity of the proteins was confirmed by immunoblotting (not shown). (B) Anionic chromatography of N-SSc5D recovered from TCS samples and further purification. (C) N-SSc5D fractions 43–46 were collected and run on 7.5% SDS-PAGE and stained with Coomassie. N-SSc5D expression was confirmed by immunoblotting (bottom). [file Image_1.TIF]

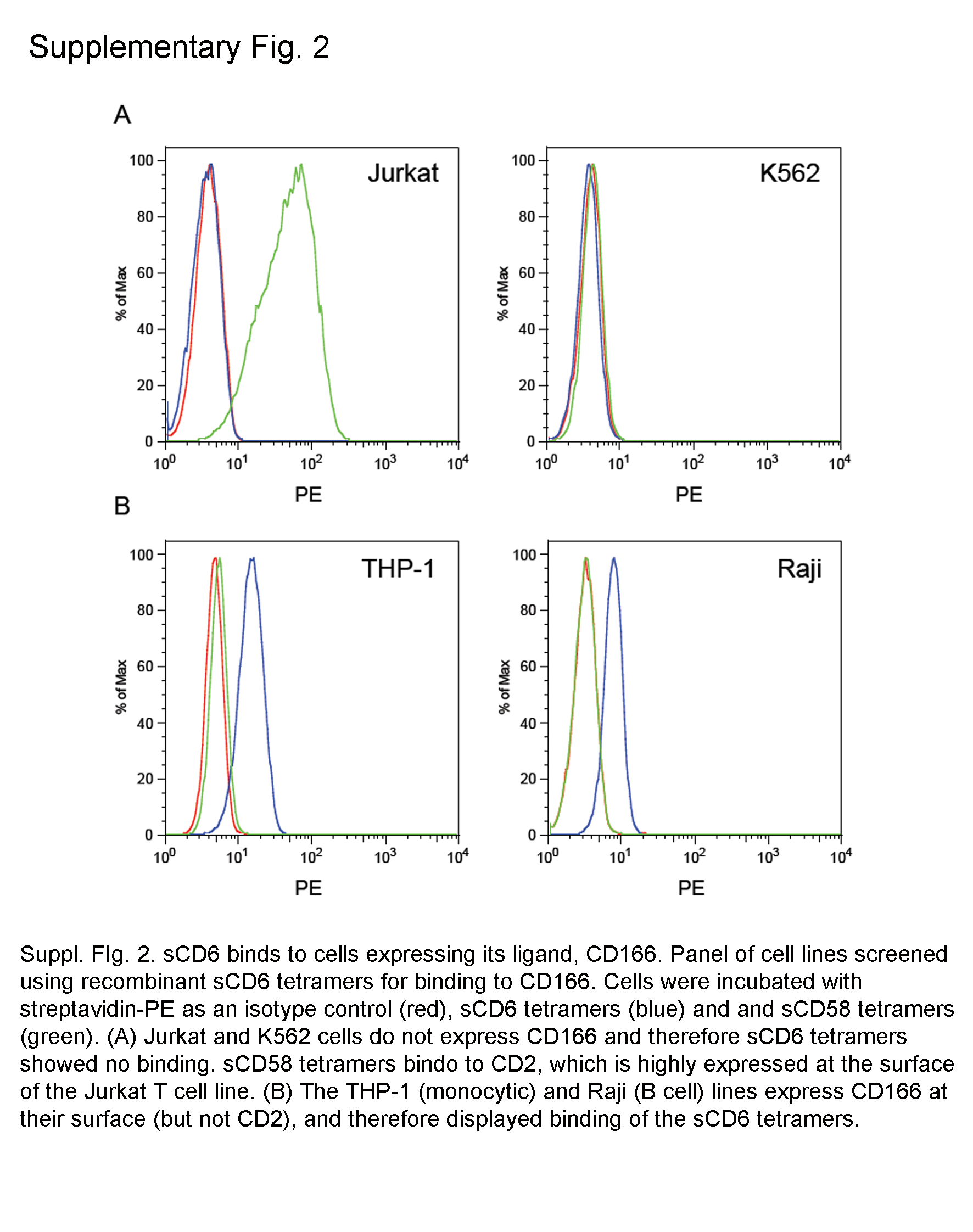

Supplement: Figure S2 — sCD6 binds to cells expressing its ligand, CD166. Panel of cells screened using recombinant sCD6 tetramers for binding to CD166. Cells were incubated with streptavidin-PE as an isotype control (red), sCD6 tetramers (blue), and sCD58 tetramers (green). (A) Jurkat and K562 cells do not express CD166, and therefore sCD6 tetramers showed no binding. sCD58 tetramers bind to CD2, which is highly expressed at the surface of the Jurkat T cell line. (B) The THP-1 (monocytic) and Raji (B cell) lines express CD166 at their surface (but not CD2) and therefore displayed binding of the sCD6 tetramers. [file Image_2.TIF]
